# Identification of a new class of natural product MDM2 inhibitor: *In vitro* and *in vivo* anti-breast cancer activities and target validation

**DOI:** 10.18632/oncotarget.3098

**Published:** 2014-12-30

**Authors:** Jiang-Jiang Qin, Wei Wang, Sukesh Voruganti, Hui Wang, Wei-Dong Zhang, Ruiwen Zhang

**Affiliations:** ^1^ Department of Pharmaceutical Sciences, School of Pharmacy, Texas Tech University Health Sciences Center, Amarillo, TX, USA; ^2^ Cancer Biology Center, School of Pharmacy, Texas Tech University Health Sciences Center, Amarillo, TX, USA; ^3^ Institute for Nutritional Sciences, Shanghai Institutes for Biological Sciences, Chinese Academy of Sciences, Shanghai, PR China; ^4^ School of Pharmacy, Shanghai Jiao Tong University, Shanghai, PR China

**Keywords:** MDM2 inhibitor, p53-independent, breast cancer, lung metastasis

## Abstract

The *MDM2* oncogene has been suggested as a molecular target for treating human cancers, including breast cancer. Most MDM2 inhibitors under development are targeting the MDM2-p53 binding, and have little or no effects on cancers without functional p53, such as advanced breast cancer. The present study was designed to develop a new class of MDM2 inhibitors that exhibit anticancer activity in MDM2-dependent and p53-independent manners. The selective MDM2 inhibitors were discovered by a computational structure-based screening, yielding a lead compound, termed JapA. We further found that JapA inhibited cell growth, decreased cell proliferation, and induced G2/M phase arrest and apoptosis in breast cancer cells through an MDM2-dependent mechanism, regardless of p53 status. It also inhibited the tumor growth and lung metastasis in breast cancer xenograft models without causing any host toxicity. Furthermore, JapA directly bound to MDM2 protein and reduced MDM2 levels in cancer cells *in vitro* and *in vivo* by promoting MDM2 protein degradation and inhibiting *MDM2* transcription, which is distinct from the existing MDM2 inhibitors. In conclusion, JapA represents a new class of MDM2 inhibitor that exerts its anticancer activity through directly down-regulating MDM2, and might be developed as a novel cancer therapeutic agent.

## INTRODUCTION

Breast cancer is the leading cause of cancer death among women in the United States and worldwide [1]. Thanks to recent advances in several fronts of breast cancer research, such as the identification of risk factors, molecular mechanisms of oncogenesis, imaging and screening strategies for early detection, the development of the genetic/genomic and molecular diagnosis, as well as targeted therapy, the prognosis and survival of patients with breast cancer have been improving [2-7]. However, for most breast cancer patients with advanced disease, especially those with triple negative breast cancer (TNBC; lacking the expression of the estrogen receptor, progesterone receptor, and human epidermal growth factor receptor 2), there are few or no effective treatment options, and the prognosis remains poor [8-10]. One of the major hurdles in the treatment and prevention of breast cancer is the lack of a good understanding of the underlying mechanisms responsible for the development and progression of this disease, including its recurrence, metastasis and resistance to treatment. Increasing evidence suggests that the loss of tumor suppressors, such as p53 and BRCA, and the overexpression of oncogenes, such as *MDM2* and *NFAT1*, play important roles in the progression of breast cancer to advanced disease [11-18]. These findings provide novel molecular targets for the treatment of breast cancer.

The *MDM2* oncogene is a major negative regulator of the tumor suppressor p53 [19], and there is an MDM2-p53 feedback auto-regulatory pathway: p53 is a positive regulator of MDM2 expression, while MDM2 directly binds to p53 and represses its transcriptional activity and promotes p53 degradation [19-20]. MDM2 also exerts oncogenic activities in a p53-independent fashion [21-24]. In cancer patients with tumors harboring mutant p53 or without p53 expression, including breast cancer patients, MDM2 overexpression is still found to be involved in cancer growth and metastasis [17, 25-26]. We and others have demonstrated that MDM2 is a promising molecular target for cancer therapy [21, 24, 27-30]. To date, most small molecule inhibitors (SMIs) of MDM2 have been designed to block the MDM2-p53 interaction [31], such as Nutlin-3 [32], RITA [33], MI-219 [34], AMG232 [35], and SAR405838 [36]. These MDM2 SMIs induce apoptosis of cancer cells harboring wild-type p53, but have low or no efficacy against cancer cells containing mutant or deficient p53. Because over 60-88% of advanced breast cancer especially TNBC harbors mutant p53 [11, 37-38], no significant anticancer activity of these MDM2 SMIs is expected in these types of cancer. Therefore, new strategies to target MDM2 are desirable.

**Figure 1:**
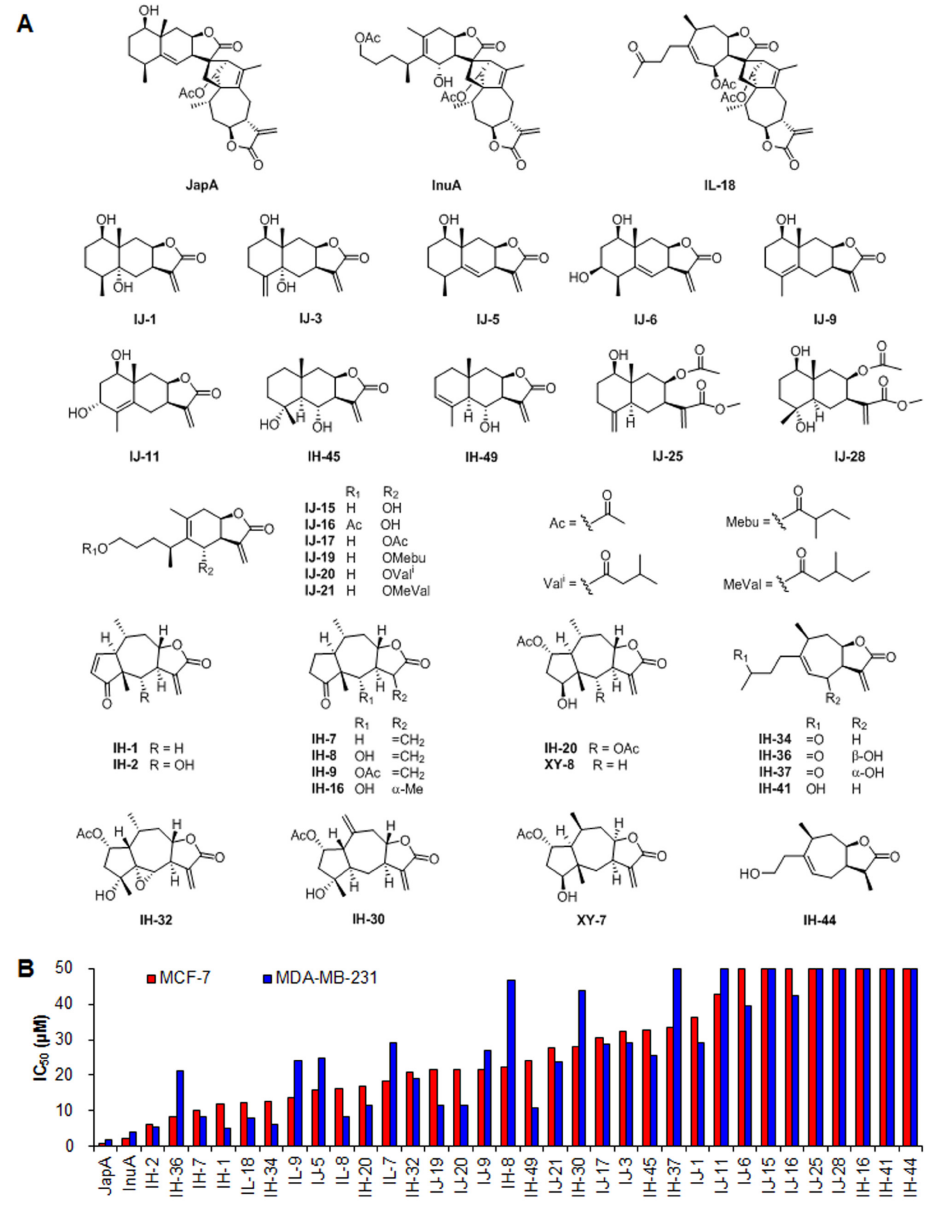
Identification of JapA and its analogs as new MDM2 inhibitors (A) The chemical structures of selected candidate compounds via a computational structure-based screening. (B) MCF-7 and MD-MBA-231 cells were treated with various concentrations of the selected compounds (0-50 μM) for 72 h, and the cell viability was analyzed using the MTT assay.

Considering that MDM2 exerts its oncogenic functions via both p53-dependent and –independent mechanisms, it is urgently needed to identify compounds that directly inhibit MDM2 and exhibit the anticancer activity, regardless of p53 status of the cancer cells. We have developed a virtual screening method to identify small molecules that have direct inhibitory effects on MDM2 [3, 39]. From our initial screening of a natural product library, we have identified a series of sesquiterpenoid and disesquiterpenoid compounds (Figure 1A) as a new class of MDM2 inhibitors. Among these potential hits, a novel C11, C3′-linked eudesmanolide-guaianolide disesquiterpenoid compound, named JapA (Figure 1A), was shown to be the most active agent. The present study was designed to investigate the *in vitro* and *in vivo* anti-breast cancer activity of JapA and the underlying molecular mechanisms of action. Our results would help demonstrate the therapeutic potentials of targeting MDM2 itself and provide a basis for further preclinical and clinical development of JapA as an anti-breast cancer agent, especially for the TNBC treatment.

## RESULTS

### Identification of JapA and its analogs as a new class of MDM2 inhibitors

In our previous studies, we have developed a computational structure-based screening method to identify compounds that specifically target MDM2 [3, 39]. The docking of virtual compounds that could bind to MDM2 protein was undertaken using the Maestro 9.0 software program (Schrodiger) [3, 39]. Based on this method, we recently performed a screening of a natural product based library and selected 35 top candidates with excellent binding affinity to MDM2 protein for further investigation (Figure 1A). These candidate compounds were further tested in more than 50 cell lines of various cancer types in our lab and breast cancer was among the most sensitive cancer types. We found that each of these compounds showed comparable cytotoxicity in MCF-7 (ER positive and p53 wild-type) and MDA-MB-231 (triple negative and p53 mutant) breast cancer cell lines (Figure 1B). In addition, α-methylene-γ-lactone group plays a crucial role in the inhibitory effects of these compounds against breast cancer cells (Figures 1A and 1B). The disesquiterpenoid compounds, *i.e.* JapA, InuA, and IL18, exhibited more potent cytotoxicity than the sesquiterpenoids (Figures 1A and 1B). JapA (Figure 1A) was selected as a lead compound based on its IC_50_ values (Figure 1B) and significant inhibitory effects on MDM2 expression in breast cancer cells.

### *In vitro* anti-breast cancer activity of lead compound JapA

**Figure 2:**
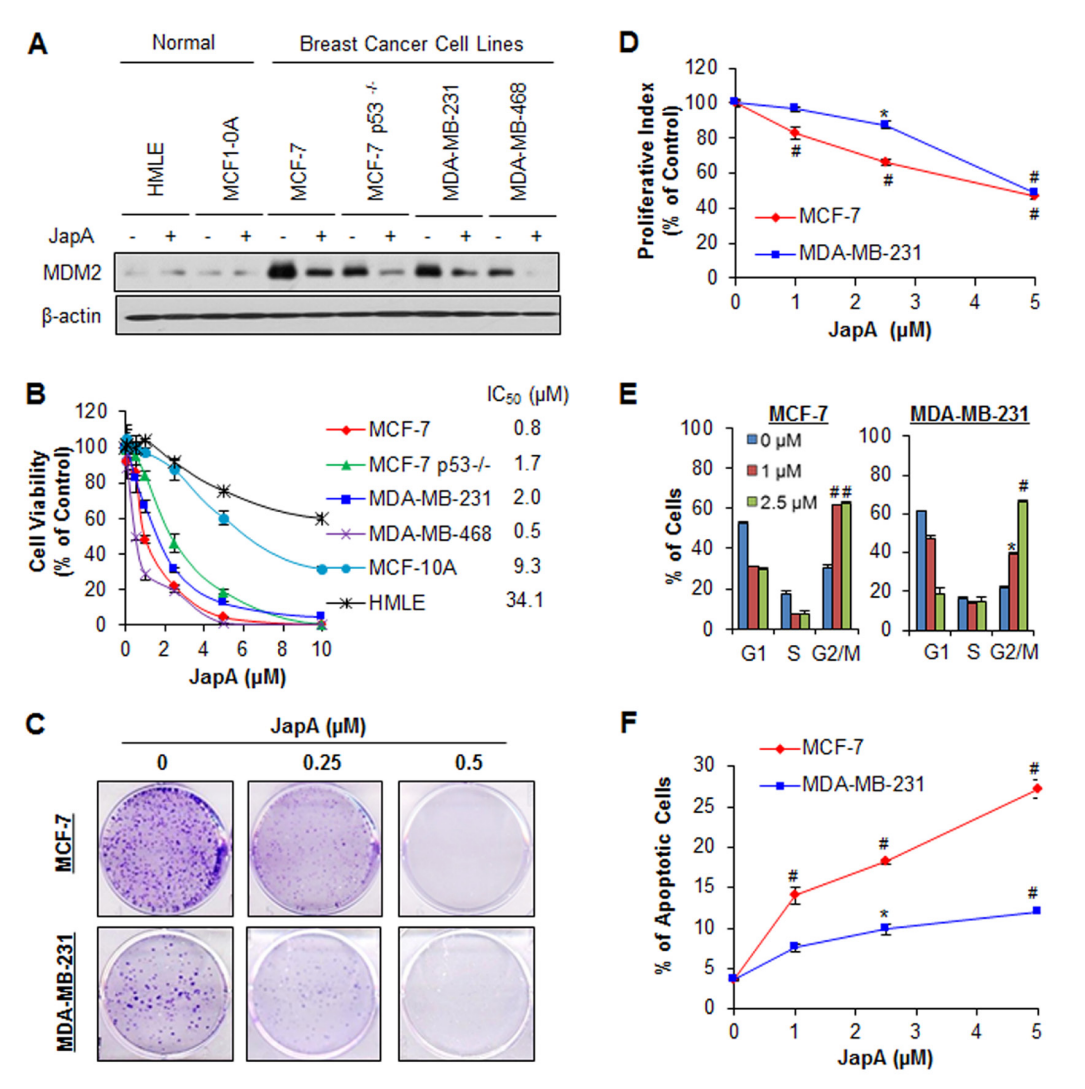
*In vivo* anti-breast cancer activity of JapA (A) Human normal breast epithelial cells and breast cancer cells were exposed to 2 μM JapA for 24 h. The protein levels of MDM2 were detected by Western blotting. The cells were further exposed to various concentrations of JapA for (B) 72 h for determination of the cell viability and IC_50_ values; (C) 24 h for the colony formation assay; (D) 24 h for the proliferation assay, where the proliferation index was calculated by comparing the proliferation of treated cells with that of untreated cells; (E) 24 h for the cell cycle distribution assay, where the cell cycle distribution was evaluated by comparing with that of untreated cells; and (F) 48 h for the cell apoptosis, which was determined by the Annexin V-FITC method. All assays were performed in triplicate (*P < 0.05 and ^#^*P* < 0.01).

The inhibitory effects of JapA on cell viability and MDM2 protein levels were confirmed in normal human breast cell lines and human breast cancer cell lines with different p53 and MDM2 statuses. As shown in Figure 2A, 2 μM JapA significantly reduced the MDM2 expression levels in MCF-7, MCF-7/p53^−/−^(p53 knockdown), MDA-MB-231, and MDA-MB-468 (p53 mutant) breast cancer cell lines. However, no apparent effect of JapA on MDM2 was observed at the same concentration in human breast epithelial MCF-10A and human mammary luminal epithelial (HMLE) cell lines. The IC_50_ values of JapA against these breast cancer cell lines ranged from 0.5 to 2.0 μM (Figure 2B). The MCF-10A and HMLE cell lines were much less sensitive to the compound than breast cancer cell lines, suggesting that JapA has a selective cytotoxicity for cancer cells and exerts its activity in an MDM2-dependent fashion, regardless of the p53 status.

JapA also inhibited the cell colony formation in a concentration-dependent manner in the MCF-7 and MDA-MB-231 cell lines (Figure 2C). Similar to the effects on cell viability, JapA inhibited the proliferation of cancer cell lines (Figure 2D); at a concentration of 5 μM, JapA inhibited the proliferation by about 52% (P < 0.01) and 51% (P < 0.01) in MCF-7 and MDA-MB-231 cells, respectively. In addition to inhibiting cell proliferation, JapA treatment arrested cells in G2/M phase, with initial effects beginning at the 1 μM concentration (P < 0.01) (Figure 2E). We also observed that JapA induced apoptosis in both breast cancer cell lines, regardless of their p53 status (Figure 2F). In the MCF-7 and MDA-MB-231 cells, 5 μM JapA increased the apoptotic index by 7.6-fold (P < 0.01) and 3.2-fold (P < 0.01), respectively, compared to control cells.

### *In vivo* efficacy of lead compound JapA in breast cancer xenograft models

**Figure 3:**
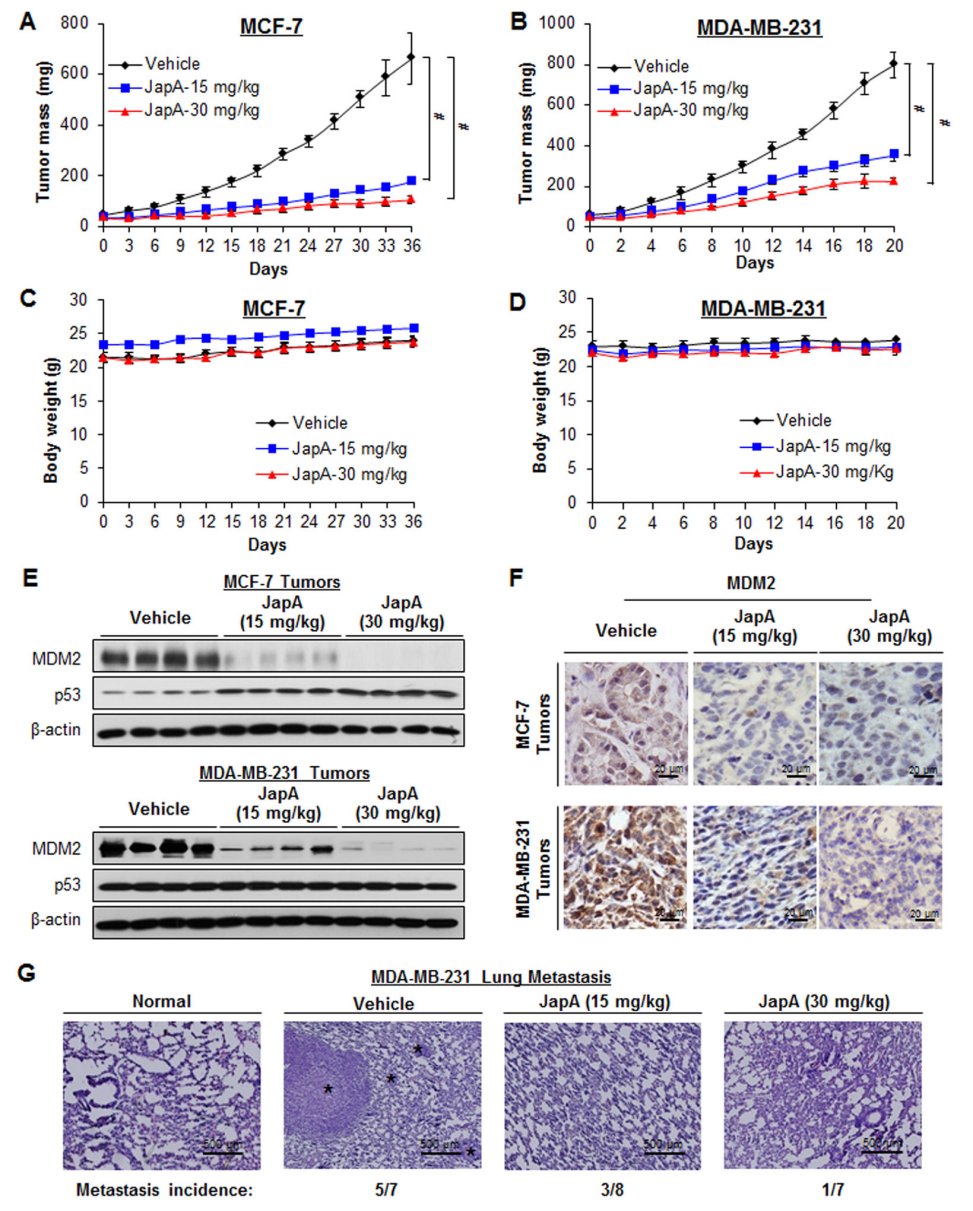
*In vivo* anti-breast cancer activity of JapA JapA was administered by i.p. injection to nude mice bearing MCF-7 (A) or MDA-MB-231 (B) xenograft tumors. Animals were monitored for changes in body weight as a surrogate marker for toxicity in the MCF-7 (C) and MDA-MB-231 (D) xenograft models. At the end of the experiments, tumors were analyzed for their protein expression of MDM2 and p53 by (E) Western blotting (each lane represents a different tumor sample) and (F) immunohistochemistry (scale bar, 20 μm). (G) Lung tissues from mice bearing MDA-MB-231 xenograft tumors were analyzed by H&E staining (scale bar, 500 μm). The numbers of mice with lung metastasis are shown. The black asterisk indicates areas of breast cancer cell invasion (^#^*P* < 0.01).

Nude mice bearing MCF-7 and MDA-MB-231 xenograft tumors were treated with JapA by i.p. injection at doses of 15 or 30 mg/kg/d, 5 d/week, for 5 weeks and 3 weeks, respectively. Dose-dependent tumor growth inhibition was observed in both models. As shown in Figure 3A, two dose levels of JapA (15 and 30 mg/kg) inhibited MCF-7 xenograft tumor growth by about 77% and 87%, respectively (P < 0.01). Same doses caused 56% and 72% inhibition in MDA-MB-231 xenograft tumor model, respectively (P < 0.01) (Figure 3B). Of note, there were no remarkable changes in the average body weights in either model, suggesting that the treatment did not lead to overt toxicity (Figures 3C and 3D).

To further demonstrate the inhibitory effects of JapA on MDM2 *in vivo*, we evaluated the protein expression levels of MDM2 and p53 in the xenograft tumors. As shown in Figure 3E, the protein levels of MDM2 were significantly reduced in a dose-dependent manner in both models, whereas the protein expression of wild-type p53 in the MCF-7 xenograft model was increased by JapA. No significant change in the levels of mutant p53 in the MDA-MB-231 tumors was observed. These results were confirmed by an immunohistochemical analysis in both models (Figure 3F).

**Figure 4:**
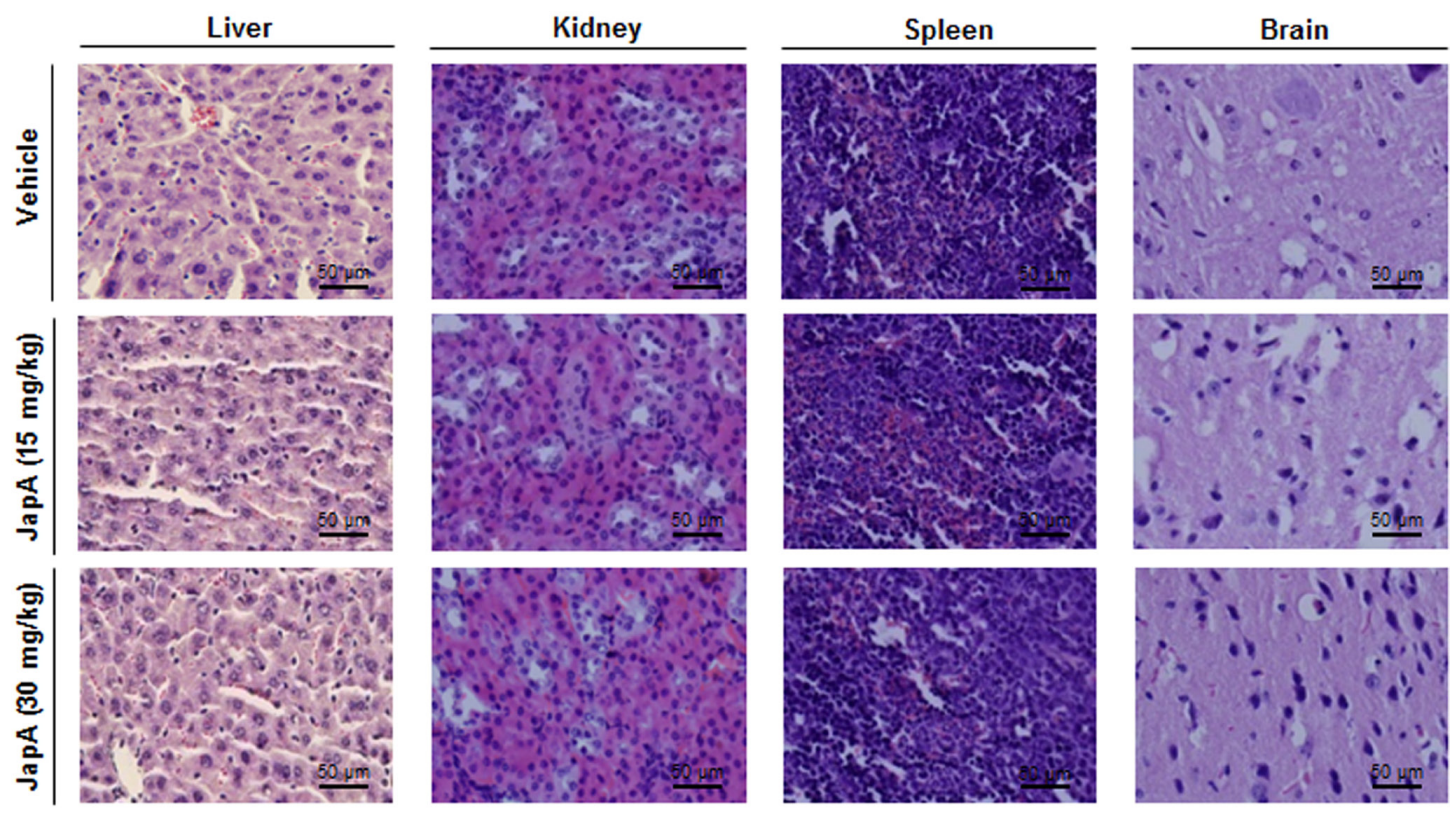
No host toxicity caused by JapA treatment JapA was administered for 3 weeks by i.p. injection to nude mice bearing MDA-MB-231 xenograft tumors. At the end of the experiments, H&E staining of the paraffin-embedded sections of various tissues (liver, kidney, spleen, and brain) obtained from mice were performed to assess whether there were any abnormalities caused by the treatment (scale bar, 50 μm).

Advanced human breast cancer, such as TNBC, is characterized by distant metastasis to various organs, especially the lungs and bone [9-11]. In MDA-MB-231 TNBC xenograft model, we also examined the development of distant metastasis and found that JapA inhibited lung metastasis, with the incidence of lung metastasis being 5/7, 3/8, and 1/7 in the vehicle-, 15 mg/kg/d JapA-, and 30 mg/kg/d JapA-treated groups, respectively (Figure 3G). There were no significant differences in the histological findings among the treatment and control groups in any of the other tissues examined (liver, kidney, spleen and brain) (Figure 4), indicating that JapA may not cause toxicity in these organs at the effective doses.

### Mechanism of action of MDM2 inhibition by lead compound JapA

**Figure 5:**
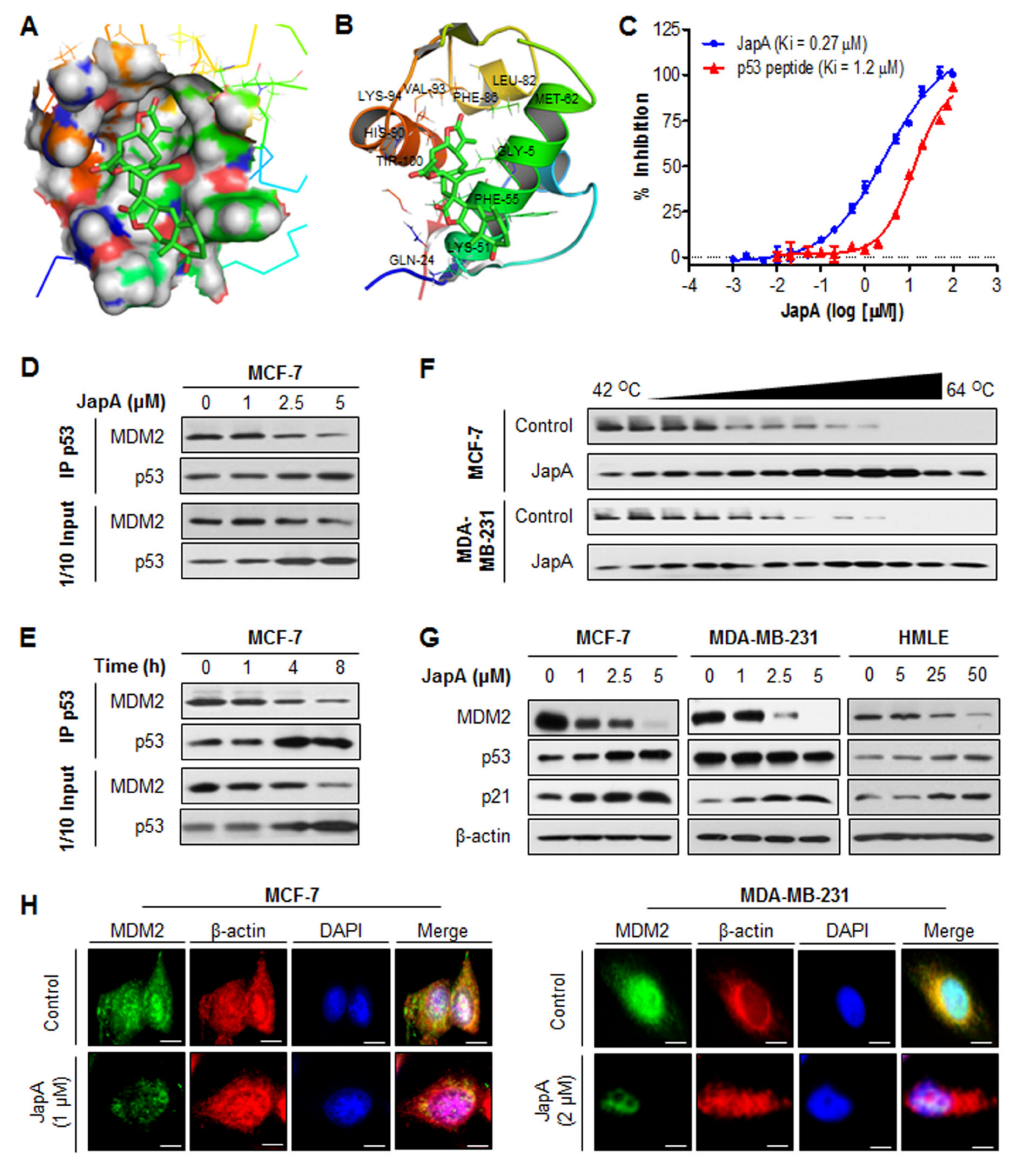
The binding of JapA to MDM2 protein (A) The binding site and orientation of JapA in the hydrophobic groove of MDM2. The protein is rendered as surface; JapA is rendered in green, with the atoms important for binding highlighted in red. (B) A model showing the interaction of JapA with MDM2. MDM2 is rendered as a cartoon, while the residues in contact with JapA are rendered as sticks. JapA is colored green, with atoms important for binding highlighted in red. (C) Competitive binding to recombinant human MDM2 proteins using fluorescence polarization-based binding assay. A natural p53 peptide was used as a positive control. (D, E) MCF-7 cells were treated with (D) various concentrations of JapA for 4 h or (E) 2 μM JapA for various times. The effect of JapA on MDM2-p53 interaction and the expression of MDM2 and p53 were determined by immunoprecipitation and Western blotting. (F) MCF-7 and MDA-MB-231 cells were exposed to 5 μM of JapA for 3 h, followed by cellular thermal shift assay. The target engagement of JapA to MDM2 protein in breast cancer cells were detected by Western blotting. (G) The cells were exposed to various concentrations of JapA for 24 h for the expression of MDM2, p53, and p21. (H) The cells were treated with JapA (1 and 2 μM, respectively, for MCF-7 and MDA-MB-231 cells) or vehicle for 24 h, followed by immunofluorescence detection (scale bar, 5 μm). β-actin and DAPI were used as internal references. All assays were performed in triplicate.

To explore the underlying molecular mechanisms responsible for JapA-induced MDM2 inhibition, a molecular modeling study of JapA and MDM2 was carried out. As shown in Figures 5A and 5B, JapA could bind in the hydrophobic pocket of MDM2 that is occupied by key p53 residues (Phe19, Trp23, and Leu26). We further evaluated the binding affinity of JapA to MDM2 protein using a fluorescence polarization (FP)-based binding assay. The results indicated that JapA bound to MDM2 protein with a *K*_i_ value of 0.27 μM (Figure 5C), showing a higher binding affinity than a p53 peptide (residues 16-27, *K*_i_ = 1.2 μM). JapA was further evaluated for its ability to block the intracellular MDM2-p53 interaction. Although the immunoprecipitation assays showed the dissociation of MDM2-p53 complex by JapA in MCF-7 cells, this compound also caused significant down-regulation of MDM2 and up-regulation of p53 in dose- and time-dependent manners (Figures 5D and 5E), which is different from the existing MDM2 inhibitors. We next sought to determine whether JapA directly binds to MDM2 protein in intact cells by employing the cellular thermal shift assay. Using this target engagement assay, we observed that JapA efficiently bound to MDM2 protein in both MCF-7 and MDA-MB-231 cell lines (Figure 5F). We further demonstrated that JapA decreased the MDM2 protein expression in breast cancer cells and normal breast cells in a concentration-dependent manner, independent of p53 (Figure 5G). However, 5 μM JapA failed to reduce MDM2 protein level in normal HMLE cells, indicating a lower response of normal cells to JapA treatment (Figure 5G). In addition, this MDM2 inhibition resulted in p53 activation in MCF-7 and HMLE cells and an increase in the expression levels of p21 in all three cell lines (Figure 5G). The JapA-induced down-regulation of the MDM2 protein was confirmed by immunofluorescence detection in breast cancer cell lines. The staining for MDM2 in both nucleus and cytoplasm was markedly decreased by JapA in the treated cells in comparison with control cells (Figure 5H).

### Effects of lead compound JapA on MDM2 protein stability

**Figure 6:**
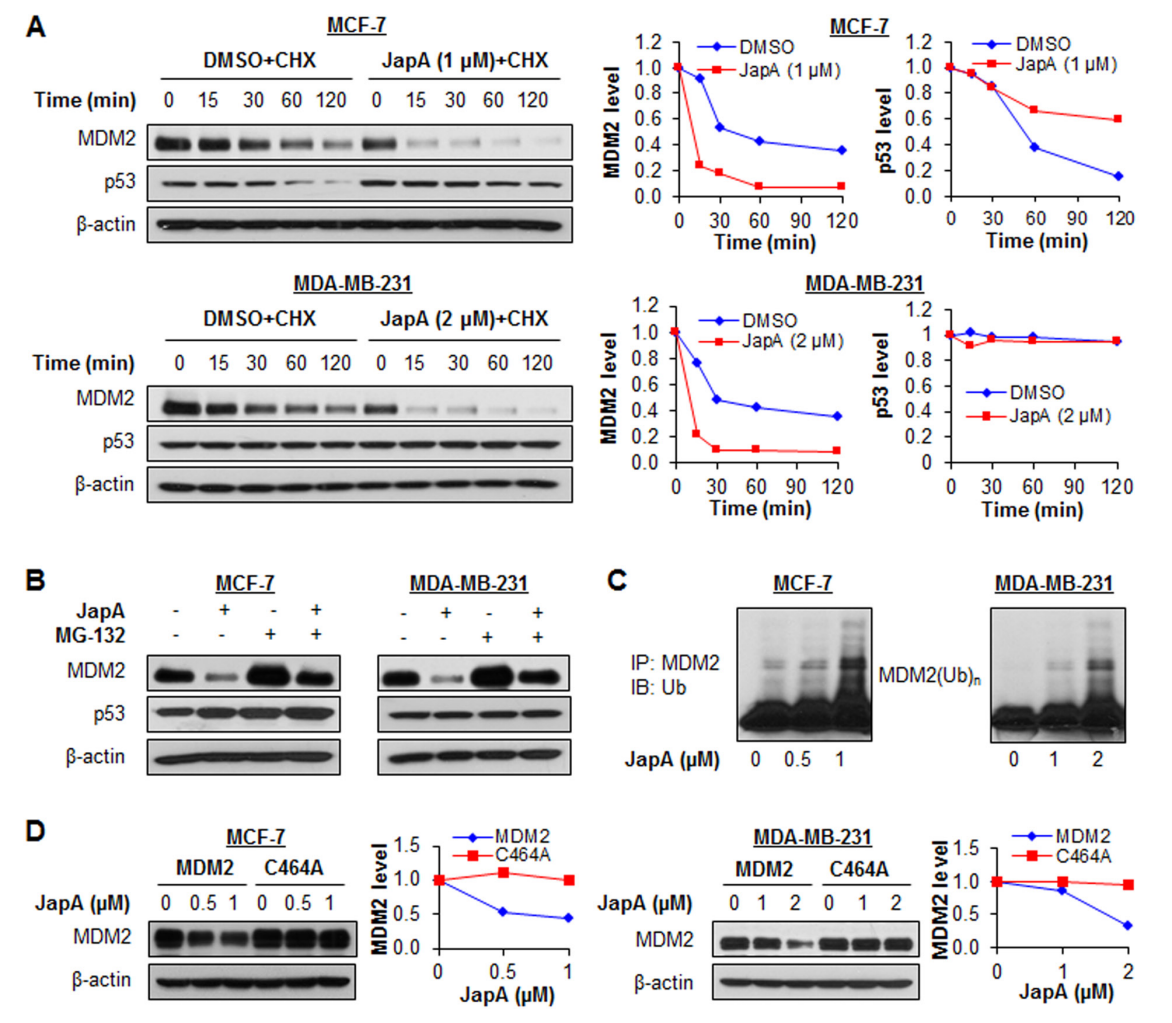
Effects of JapA on MDM2 protein stability (A) MCF-7 and MDA-MB-231 cells were treated with JapA or vehicle, followed by exposure to a protein synthesis inhibitor, cycloheximide (CHX, 15 μg/mL). The MDM2 and p53 protein levels were detected by Western blotting at the indicated times after exposure to CHX. Graphs (right) show the quantification of the immunoblotting data. (B) The cells were treated with JapA (1 and 2 μM, respectively, for MCF-7 and MDA-MB-231 cells) or vehicle for 24 h, then were exposed to MG-132 (25 μM), a proteasome inhibitor, for an additional 6 h. The protein levels of MDM2 and p53 were detected by Western blotting. (C) The cells were co-transfected with MDM2 and ubiquitin plasmids, followed by treatment with JapA for 24 h. Cell lysates were subjected to immunoprecipitation with an MDM2 antibody. The ubiquitinated MDM2 was detected using an anti-ubiquitin antibody. (D) The cells were transfected with a wild-type MDM2 plasmid or a mutant MDM2 plasmid (C464A) without E3 ligase activity, followed by exposure to JapA for 24 h, and the MDM2 levels were detected by Western blotting. Graphs (right) show the quantification of the immunoblotting data. All of the experiments were repeated three times.

We next explored how JapA reduced the MDM2 protein level. In the presence of cycloheximide (CHX), a protein synthesis inhibitor, JapA increased the degradation rate of the MDM2 protein in both MCF-7 and MDA-MB-231 cells (Figure 6A). The half-life of wild-type p53 was prolonged in MCF-7 cells, while no significant change in the half-life of the mutant p53 protein was observed in the MDA-MB-231 cells (Figure 6A). However, treatment with MG-132, a proteasome inhibitor, reduced the MDM2 protein degradation by JapA in both cell lines (Figure 6B), suggesting that the JapA-induced MDM2 degradation is proteasome-dependent. These results were paralleled by the observation that JapA induced MDM2 ubiquitination (Figure 6C). Taken together, these results indicate that JapA treatment leads to increased MDM2 degradation through the ubiquitin-proteasome pathway. Considering that MDM2 is an E3 ubiquitin ligase, and that its degradation largely depends on autoubiquitination [40], we wanted to delineate whether JapA induces MDM2 autoubiquitination. To address this possibility, MCF-7 and MDA-MB-231 cells were transfected with MDM2 or an MDM2 mutant (C464A) without E3 ubiquitin ligase activity, followed by JapA treatment. As shown in Figure 6D, JapA induced the degradation of wild-type MDM2, but not mutant MDM2 (C464A). These findings led us to conclude that JapA destabilizes the MDM2 protein by inducing its autoubiquitination and proteasomal degradation in breast cancer cells.

### Effects of lead compound JapA on *MDM2* transcription

**Figure 7:**
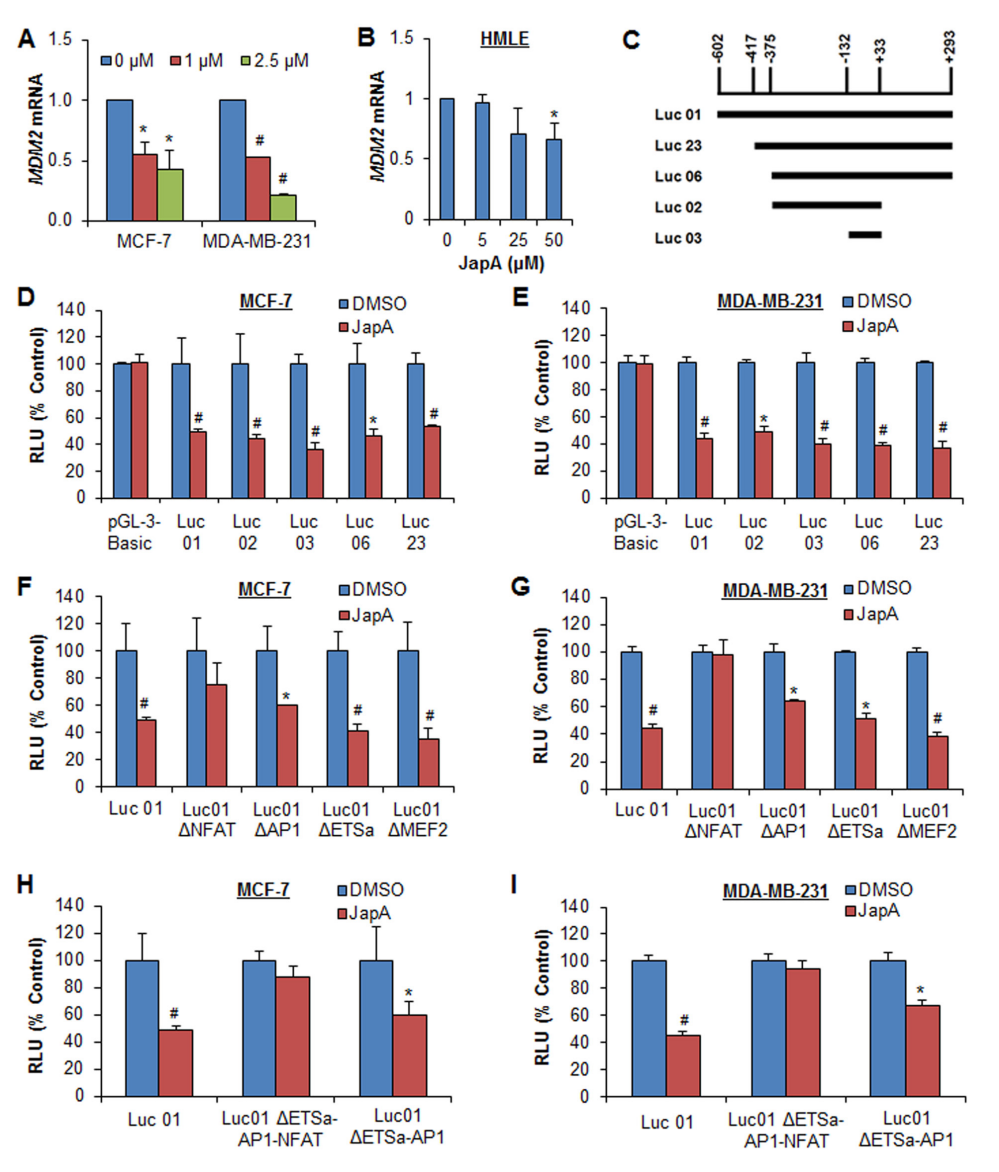
Effects of JapA on *MDM2* transcription (A) MCF-7 and MDA-MB-231 cells were treated with JapA (0, 1 and 2.5 μM) for 24 h. (B) Normal breast HMLE cells were treated with JapA (0, 5, 25 and 50 μM) for 24 h. The relative levels of *MDM2* mRNA were normalized to those of *GAPDH* mRNA. (C) The structures of the full length and deleted *MDM2* P2 promoters. (D, E) The cells were transfected with full length or deleted *MDM2* P2 promoters, or the corresponding empty vector (pGL3-Basic), for 12 h, followed by treatment with JapA (1 and 2 μM, respectively, for MCF-7 and MDA-MB-231 cells) for an additional 24 h. (F, G) The cells were transfected with full length or site-mutated *MDM2* P2 promoters for 12 h, followed by treatment with JapA (1 and 2 μM, respectively) for an additional 24 h. (H, I) The cells were transfected with the *MDM2* P2 promoter or *MDM2* P2 promoters with a double or triple mutation for 12 h, followed by treatment with JapA (1 or 2 μM, respectively) for 24 h. The MDM2 luciferase activities were detected using the Dual-Luciferase Reporter Assay System (*P < 0.05 and ^#^*P* < 0.01).

We next explored whether JapA affects MDM2 at the transcriptional level. As shown in Figure 7A, JapA reduced the *MDM2* mRNA levels in breast cancer cells in a concentration-dependent manner, regardless of the p53 status. Although JapA down-regulated the *MDM2* mRNA level in normal breast HMLE cells, it had no significant effect at 5 μM, which is an effective concentration in breast cancer cells (Figure 7B). We then demonstrated that JapA inhibited the *MDM2* P2 promoter activity by using a full-length reporter (Luc01) and various deletions (Figure 7C). The results showed decreased luciferase activities for all of the reporters following JapA treatment in both cell lines (Figures 7D and 7E), with the shortest deletion, Luc 03 (−132 to +33), still showing a response to JapA. The luciferase activity of the *MDM2* reporter (Luc01) was decreased 64% (P < 0.01) and 63% (P < 0.01) by JapA in MCF-7 and MDA-MB-231 cell lines, respectively; there were no apparent changes in the cells transfected with the corresponding empty vector reporter. To identify the JapA-responsive site on the *MDM2* P2 promoter, several transcription factor sites-mutated P2 luciferase vectors, *i.e.,* with ETSα, AP1, MEF2, and NFAT mutations, were transfected into MCF-7 and MDA-MB-231 cells, followed by JapA treatment. As shown in Figures 7F and 7G, the NFAT mutation effectively eliminated the response to JapA, whereas the other three mutant vectors retained the capacity to respond to the JapA treatment. Further studies using vectors with double mutations (ΔAP1-ETSα) and triple mutations (ΔAP1-ETSα-NFAT) also supported that NFAT is mainly responsible for JapA's inhibitory effects on the *MDM2* P2 promoter (Figures 7H and 7I).

### Effects of MDM2 overexpression and knockdown on JapA's activity

**Figure 8:**
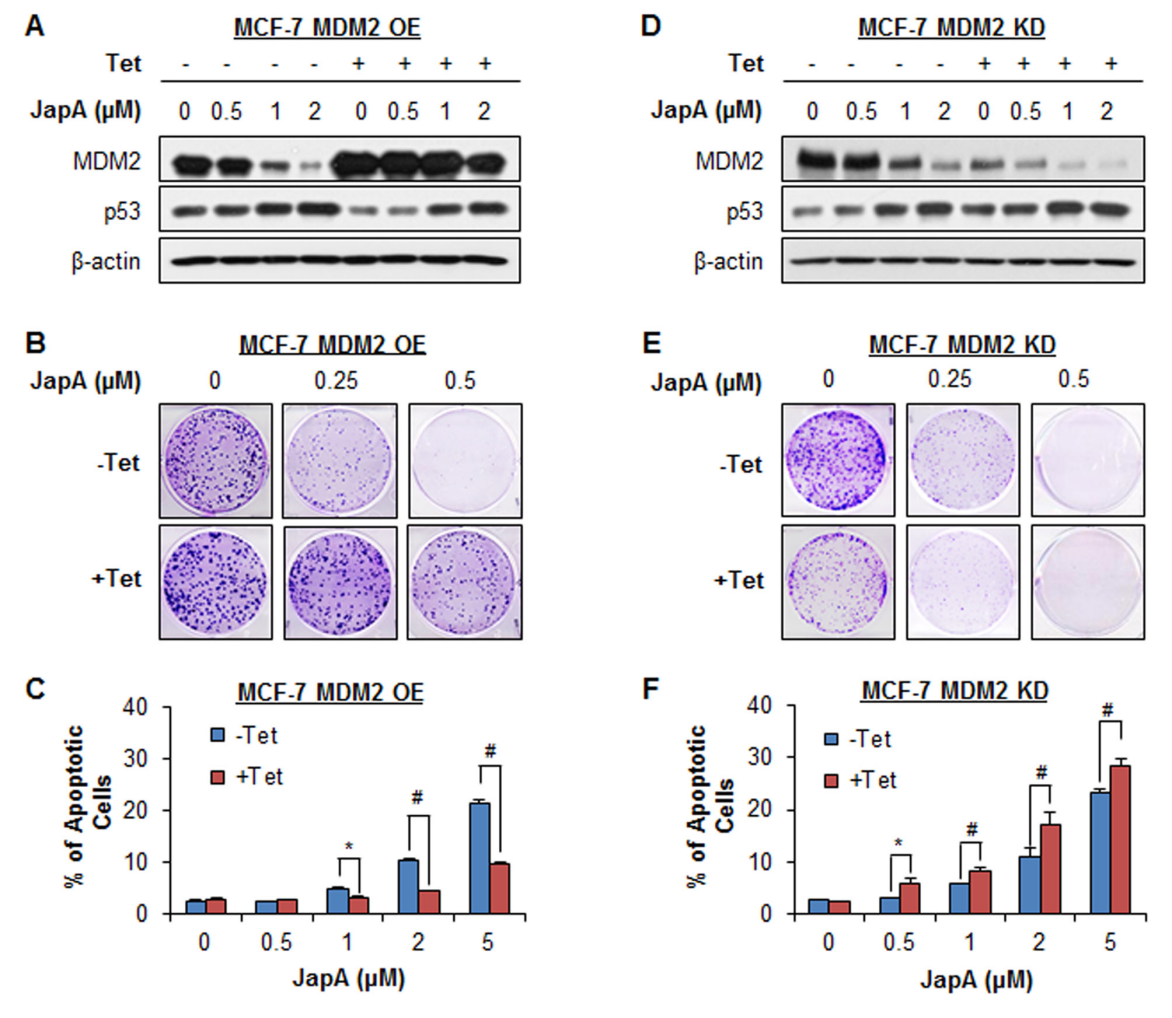
Effects of MDM2 overexpression and knockdown on JapA's activity The inducible MDM2 overexpression and knockdown MCF-7 cells were incubated with (+Tet; 1 μg/mL) or without tetracycline (−Tet) for 24 h and then treated by various concentrations of JapA for (A, D) 24 h for the expression of MDM2 and p53; (B, E) 24 h for the colony formation assay; and (C, F) 48 h for the cell apoptosis, which was determined by the Annexin V-FITC method. All assays were performed in triplicate (*P < 0.05 and ^#^*P* < 0.01).

**Figure 9:**
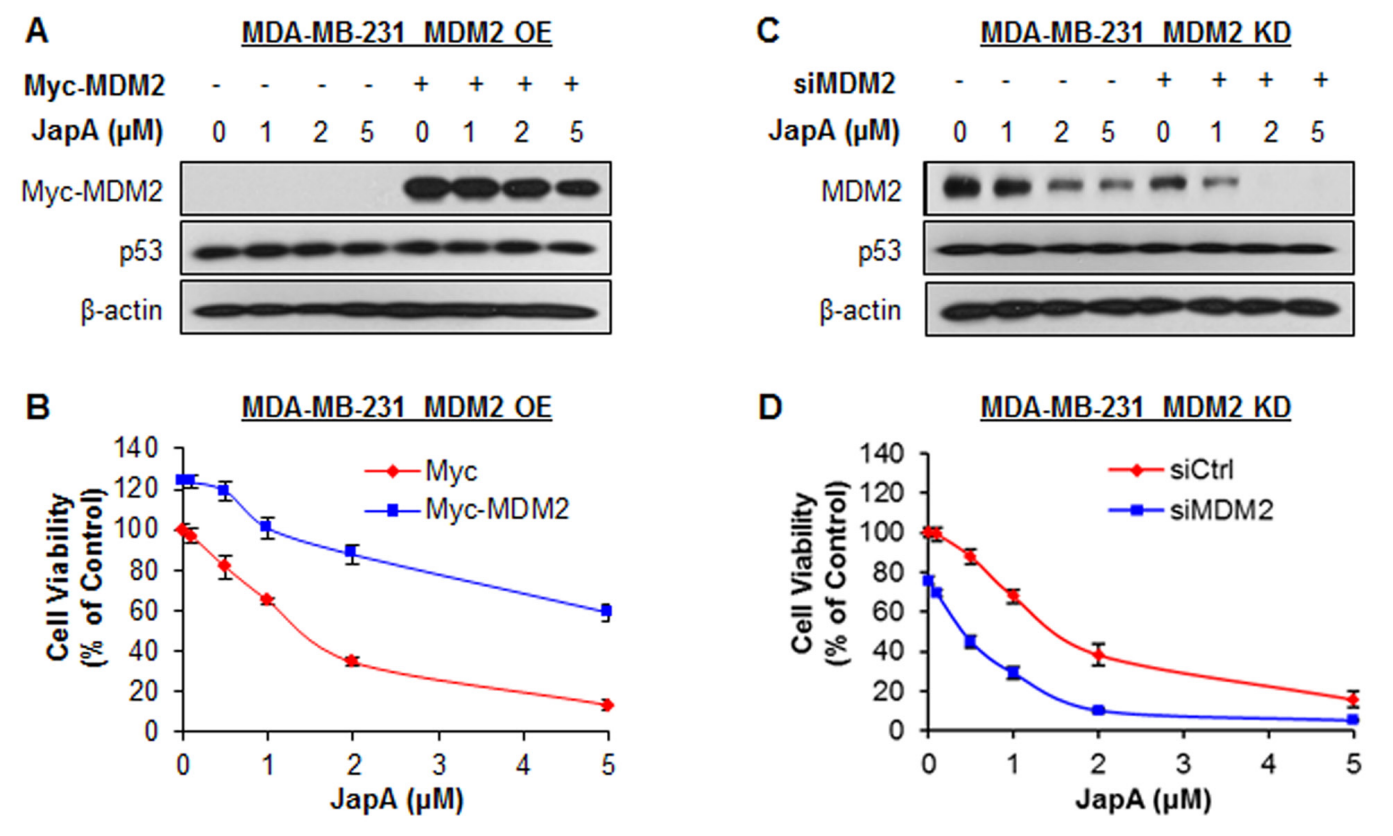
Effects of MDM2 overexpression and knockdown on JapA-induced cell death (A, B) MDA-MB-231 cells were transfected with a Myc plasmid or a Myc-MDM2 plasmid for 24 h and then treated with various concentrations of JapA for (A) 24 h for the expression levels of Myc-MDM2 and p53, where Myc-MDM2 and p53 were detected by Western blotting using antibodies against Myc and p53, respectively; and (B) 72 h for determination of the cell viability. (C, D) MDA-MB-231 cells were transfected with MDM2 siRNA or the respective control siRNA for 36 h and then treated with various concentrations of JapA for (C) 24 h for expression levels of MDM2 and p53, where MDM2 and p53 were detected by Western blotting using antibodies against MDM2 and p53, respectively; and (D) 72 h for determination of the cell viability. All assays were performed in triplicate.

To demonstrate the importance of MDM2 in JapA's anticancer activity, we further tested the inhibitory effects of JapA on inducible MDM2 overexpression (OE) and knockdown (KD) MCF-7 cell lines (p53 wild-type), in comparison with their corresponding parental cell lines. In inducible MDM2 OE cells, Tet treatment resulted in a 2.6-fold OE of MDM2 protein expression (Figure 8A). Tet-induced MDM2 OE reduced the effects of JapA on MDM2 protein expression and p53 activation (Figure 8A), colony formation (Figure 8B), and cell apoptosis (Figure 8C). In inducible MDM2 KD cells, Tet treatment induced 76% KD of MDM2 protein expression. The effects of JapA on MDM2 protein expression and p53 activation (Figure 8D), colony formation (Figure 8E), and cell apoptosis (Figure 8F) were enhanced by Tet-induced MDM2 KD. We further demonstrated the role of MDM2 in JapA's activity in MDA-MB-231 cells (p53 mutant). As shown in Figures 9A and 9B, the transient transfection of a Myc-MDM2 plasmid resulted in MDM2 OE in MDA-MB-231 cells (Figure 9A), which increased the cell growth and reduced the cytotoxicity of JapA in the cells (Figure 9B). As shown in Figures 9C and 9D, the transient transfection of MDM2 siRNA caused approximately 72% KD of MDM2 protein expression (Figure 9C), decreased the cell growth, and strengthened the inhibitory effects of JapA on cell viability in MDA-MB-231 cells (Figure 9D). Taken together, these results indicated that MDM2 plays a critical role for JapA-induced anti-breast cancer activity.

## DISCUSSION

In the present study, we identified a new MDM2 inhibitor JapA and investigated its *in vitro* and *in vivo* anti-breast cancer activities and molecular mechanism of action. We have made at least five novel discoveries in this study. First, JapA and its analogs are a new class of MDM2 inhibitors that directly target MDM2 at both transcriptional and post-translational levels. These compounds have comparable cytotoxicity against breast cancer cells with different genetic backgrounds (p53 wild-type or mutant; ER positive or TNBC), indicating a p53-independent and ER-independent mechanism of action. Second, JapA selectively inhibits breast cancer cell growth, reduces the cell colony formation and proliferation, arrests cells in the G2/M phase, and induces apoptosis through an MDM2-dependent mechanism, independent of p53 status. Third, JapA suppresses breast tumor growth and lung metastasis and inhibits MDM2 expression *in vivo*, regardless of the p53 status. Fourth, JapA specifically binds to MDM2 protein and has a better binding affinity than p53 peptide, and MDM2 expression is critical for JapA's anti-breast cancer activity as indicated in our assays using MDM2 overexpression and knockdown breast cancer cells. Finally, JapA inhibits MDM2 through blocking MDM2-p53 interaction, promoting MDM2 protein degradation, and inhibiting *MDM2* transcription, which is distinct from the MDM2 inhibitors under development. These results not only demonstrate the therapeutic potential of JapA as an anti-breast cancer agent, but also support the notion that targeting MDM2 itself is a promising therapeutic strategy for advanced breast cancer.

There is an increasing interest in developing MDM2 inhibitors for cancer therapy. We and others have demonstrated that there are at least three strategies to target MDM2. First, block the MDM2-p53 interaction to release p53 from MDM2 and activate the p53 pathway in cancer cells [32-36, 41]. Second, inhibit MDM2′s E3 ligase activity to stabilize p53 and activate the p53 pathway, such as JNJ-26854165 [42]. Third, directly inhibit MDM2 expression to modulate both the p53-dependent and –independent pathways [24]. Both the first and second classes of MDM2 inhibitors require wild-type p53 expression in cancer cells. However, the majority of breast cancers contains mutant p53 and has high levels of MDM2 [11, 37-38]; such cancers are less or unresponsive to these types of MDM2 inhibitors. Therefore, we designed this study to identify compounds that have direct inhibitory effects on MDM2 for the treatment of cancer.

In the present study, JapA was demonstrated as a novel and specific MDM2 inhibitor using several assays. First, JapA showed stronger inhibitory effects on breast cancer cell growth and better specificity in targeting MDM2, in comparison with other candidate compounds. Second, JapA exhibited its anticancer activity in MDM2-dependent and p53-independent manners, as shown in normal breast cell lines and breast cancer cell lines with different MDM2 and p53 statuses. Our studies suggested that the cell lines containing higher endogenous expression levels of MDM2 have a better cell response to JapA, which contributes to the specificity of JapA in inhibiting cancer cells. Third, JapA specifically bound to the MDM2 protein as demonstrated in several assays, including molecular docking, FP-based binding assay, and cellular thermal shift assay. Fourth, the underlying mechanisms of action of JapA-induced MDM2 inhibition are significantly different from those of existing MDM2 inhibitors. Our results indicated that JapA inhibited the MDM2-p53 binding and destabilized MDM2 protein by promoting MDM2 auto-ubiquitination and proteasomal degradation. It was also found that JapA inhibited *MDM2* transcription in an NFAT-dependent manner. Fifth, consistent with antisense MDM2 inhibitors, JapA increased the expression level of p21, which has been strongly implicated in tumor initiation and progression, independent of p53 [43-44]. Finally, MDM2 plays a crucial role in the anticancer activity of JapA, as shown in MDM2 overexpression and knockdown breast cancer cell lines (both p53 wild-type and mutant).

Of note, our studies indicated that JapA occupies the hydrophobic pocket of MDM2 protein, inhibits the interaction between MDM2 and p53, and destabilizes MDM2 protein. However, it remains unknown that if the binding of JapA to MDM2 is responsible for the rapid degradation of MDM2. It is possible that JapA also binds to other domains of MDM2 or targets other upstream regulators of MDM2. It is known that the oncogene *MDMX*, a closely related homolog of MDM2, is another negative regulator of p53 and a valid molecular target for cancer therapy [26, 29, 30]. Since MDMX has a great structural similarity to MDM2, it is also probable that MDMX may be also involved in the anticancer activity of JapA. Although our *in vitro* and *in vivo* studies have indicated a critical role of MDM2 in JapA's anticancer activity, further investigations on the identification and validation of mechanisms using cutting-edge techniques and state-of-the-art cancer models are required.

There are several reasons for selecting human breast cancer as the target disease for this new class of MDM2 inhibitor. First, MDM2 is overexpressed in human breast cancer and associated with breast cancer progression, metastasis and drug resistance [13-15]. Second, in our initial screening of various human cancer cell lines, breast cancer cell lines were among the most sensitive cancer types. Third, JapA inhibits breast cancer cell growth *in vitro* and *in vivo* through inhibiting cell proliferation and cell cycle progression and inducing apoptosis. Fourth, similar *in vitro* and *in vivo* anticancer activity was observed in the MCF-7 and MDA-MB-231 models, suggesting that JapA is a promising anti-breast cancer agent, regardless of the p53 status of the tumor. Fifth, JapA did not show significant cytotoxicity to normal breast cell lines and toxicity in mice, although the dose levels may need to be optimized. Finally, considering that the MDA-MB-231 model has been widely used as a model of TNBC and breast cancer metastasis, our results suggest that JapA will be useful to treat TNBC and metastatic breast cancer.

In summary, our present results demonstrate that JapA directly inhibits MDM2, resulting in the inhibition of breast tumor growth and metastasis, regardless of the p53 status of cancer cells. This study provides evidence supporting that directly targeting MDM2 is a promising strategy for the discovery of novel anticancer agents.

## MATERIALS AND METHODS

### Cells and culture conditions

Human breast cancer (MCF-7, MDA-MB-231, and MDA-MB-468) and non-malignant epithelial (MCF-10A) cells were obtained from the American Type Culture Collection (Rockville, MD). Human mammary luminal epithelial (HMLE) cells were obtained from Zen-Bio, Inc. (Research Triangle Park, NC). All cell culture media, except that for the MCF-10A and HMLE cells, contained 10% fetal bovine serum and 1% penicillin/streptomycin. MCF-7 and MDA-MB-231 cells were grown in Dulbecco's modified Eagle's media containing 1 mM non-essential amino acids and Earle's BSS, 1 mM sodium pyruvate and 10 mg/L bovine insulin. The MCF-7 p53^−/−^ cell line was established previously [45-46] and was grown in the same media as MCF-7 cells, but supplemented with 0.5% μg/mL puromycin (Sigma; St. Louis, MO). MDA-MB-468 cells were grown in DMEM/F-12 Ham's media (1:1 mixture). MCF-10A cells were grown in DMEM/F-12 Ham's media containing 5% horse serum, 20 ng/mL EGF, 0.5 mg/mL hydrocortisone, 100 ng/mL cholera toxin, 10 μg/mL insulin, and 1% penicillin/streptomycin. HMLE cells were grown in mammary luminal epithelial cell growth medium (Zen-Bio, Inc., NC). The inducible MDM2 overexpression (OE) and knockdown (KD) MCF-7 cell lines were established previously [47] and were grown in DMEM medium containing 10 μg/mL blasticidin and 200 μg/mL zeocin (OE cells) (Invitrogen, Grand Island, NY), or 0.5% μg/mL puromycin (KD cells) (Sigma; St. Louis, MO).

### Chemicals, reagents, antibodies, plasmids, and siRNAs

The investigated compounds, including JapA, were obtained from a natural product library established in Dr. Wei-Dong Zhang's laboratory, with their purity being >95% (confirmed by IR, ESI-MS, NMR, and HPLC/MS^n^) [48]. All chemicals and solvents were of the highest analytical grade available. Cell culture supplies and media, fetal bovine serum, phosphate-buffered saline (PBS), sodium pyruvate, non-essential amino acids, and penicillin-streptomycin were obtained from Invitrogen (Carlsbad, CA). The anti-human p53 (DO-1) antibody was from Santa Cruz Biotechnology Inc. (Dallas, TX). The anti-human MDM2 (Ab-2) and p21 (Ab-1) antibodies were from Calbiochem (Billerica, MA). The anti-human ubiquitin (6C1) and β-actin (AC-15) antibodies were from Sigma (St. Louis, MO). Goat anti-mouse IgG (H+L) and goat anti-rabbit IgG (H+L) were obtained from Bio-Rad (Hercules, CA). The human full-length and deleted *MDM2* P2 promoter reporters were kind gifts from Dr. J.P. Blaydes (Southampton General Hospital, UK). The P2 promoter reporters lacking the MEF2, NFAT, and ETS-α-AP1-NFAT binding sites were generated by site-directed mutagenesis [49]. The wild-type MDM2 and mutant MDM2 (C464A without E3 ligase activity) expression vectors were kindly provided by Dr. J. Chen (Moffitt Cancer Center, USA). MDM2 siRNA or control siRNA were from Thermo Scientific (Rockford, IL). Both plasmids and siRNAs were transfected into cells using the same protocols as reported by us earlier [3, 39].

### Assays for cell viability, colony formation, cell proliferation, cell cycle distribution, and apoptosis

Cells were treated with various concentrations of JapA, and cell viability, colony formation, cell proliferation, cell cycle distribution, and apoptosis assays were performed as described previously by us [45, 49-50].

### Virtual screening and molecular modeling

The initial virtual screening of a natural product library established in Dr. Wei-Dong Zhang's laboratory was performed as we reported previously [3, 39]. Briefly, the docking of JapA and analogs with a refined structure of MDM2 (PDB: 4ERE) was carried out using Maestro 9.0 software program (Schrodinger). After removing the water molecules from the complex structure, the ‘Protein Preparation Wizard’ workflow was used to add the hydrogen atoms and charges during a brief relaxation using. The hydrogen bond network was then optimized and the crystal structure was minimized using the OPLS 2005 force field, with the maximum RMSD value of 0.3Å. The grid-enclosing box was centered on the ligand AM-8553 in the refined crystal structure and was defined so as to enclose residues located within 14 Å from the ligand. In the above step, a scaling factor of 1.0 was set to van der Waals radii with a partial atomic charge of 0.25 to soften the nonpolar parts of the receptor. In this study, the three-dimensional structures of all compounds were generated using a Ligprep module. The extra precision (XP) approaches were adopted successively.

### Assays for intracellular MDM2-p53 interaction

The effect of JapA on intracellular MDM2-p53 interaction was determined as reported previously [33-34]. Briefly, MCF-7 cells were treated with various concentrations of JapA (0, 1, 2.5 and 5 μM) for 4 h or exposed to 2 μM JapA for various times (0, 1, 4, and 8 h). Cell lysates were immunoprecipitated with an anti-p53 antibody at 4 ^o^C overnight. The bound proteins were purified with protein G-Sepharose beads (Sigma, St Louis, MO), resolved on SDS-PAGE, and detected by anti-p53 and anti-MDM2 antibodies. 1/10 of input cell lysates was analyzed by Western blotting for the expression levels of p53 and MDM2.

### Cellular thermal shift assay

The target binding ability of the test compound in intact cells was evaluated using the cellular thermal shift assay described previously [51]. Briefly, MCF-7 and MDA-MB-231 cells were seeded into 6-cm dishes at a density of 6 × 10^5^ cells/well. After 24 h, cells were treated with or without 5 μM JapA for 3 h. After treatment, cells were harvested using trypsin, collected by centrifugation and subsequently resuspended in PBS. Equal amounts of cell suspensions were aliquoted into 12 PCR tubes and heated for 3 min to 42, 44, 46, 48, 50, 52, 54, 56, 58, 60, 62 or 64 ^o^C. Cells were then lysed by three repeated cycles of freeze-thawing. The precipitated proteins were separated from the soluble fraction by centrifugation at 17,000 g for 20 min. The collected supernatants were used for Western blotting.

### Fluorescence polarization competitive binding assay

Briefly, serial dilutions of JapA, a natural peptide (residues 16-27, QETFSDLWKLLP-NH2) and the assay buffer (100 mM potassium phosphate, pH 7.5; 100 μg/ml bovine gamma globulin; 0.02% sodium azide) with preincubated MDM2 protein (10 nM, GST-MDM2) and PMDM6-F peptide (1 nM) were added in Dynex 96-well black, round-bottom plates, and the fluorescence polarization values were measured after 3 h of incubation. For each assay, the controls included the MDM2 protein and PMDM6-F (equivalent to 0% inhibition) and only PMDM6-F peptide (equivalent to 100% inhibition). The binding affinity constants (*K*i) of the test compound and p53 peptide were calculated using a web-based computer program (http://sw16.im.med.umich.edu/software/calc_ki/) which was developed in Dr. Shaomeng Wang's lab [52-53].

### Assays for protein stability

To determine the effect of JapA on the half-life of MDM2 and p53 proteins, MCF-7 and MDA-MB-231 cells were treated with or without JapA (1 or 2 μM, respectively) for 24 h. Cycloheximide (CHX, 15 μg/mL) was then added and the cells were lysed at the indicated times. The expression levels of MDM2 and p53 were detected by western blotting. To determine the effect of JapA on MDM2 and p53 protein degradation, the cells were treated with or without JapA (1 or 2 μM, respectively) for 24 h, followed by a 6-h treatment of MG-132 (25 μM). Cell lysates were collected for the protein expression levels of both MDM2 and p53.

### Real-time quantitative PCR

Total RNA was extracted from human breast cancer cells using the Trizol reagent (Invitrogen, Grand Island, NY), and a quantitative RT-PCR analysis was performed as described previously [45, 50]. The primer sequences used for the amplification of genes were as follows: MDM2 sense: 5′-ATCATCGGACTCAGGTACA-3′; MDM2 antisense: 5′-GTCAGCTAAGGAAATTTCAGG-3′; GAPDH sense: 5′-GGAGTCCACTGGCGTCTTCAC-3′; GAPDH antisense: 5′-GAGGCATTGCTGATGATCTTGAGG-3′.

### Luciferase assay

Breast cancer cells were co-transfected with full-length or deleted human MDM2 promoter vectors with the Renilla luciferase reporter as an internal control [45, 49]. The cells were then exposed to JapA for 24 h. The luciferase activity of the MDM2 promoter reporters was determined using the Dual-Luciferase Reporter Assay System (Promega, Madison, WI), according to the manufacturer's protocol. The MDM2 reporter activity was normalized to that for the Renilla luciferase reporter.

### Immunoblotting

In the *in vitro* studies, the JapA-treated and control cells were collected and lysed in NP40 lysis buffer containing protease inhibitors (Sigma, St Louis, MO). *In vivo* tissue homogenates were prepared in NP-40 lysis buffer (100 mg tumor tissue/1 mL NP-40 buffer) for the immunoblotting analysis. The protein concentration was estimated using the Bradford reagent (Bio-Rad, Hercules, CA). Cell lysates with identical amounts of protein were fractionated by SDS-PAGE, and were transferred to Bio-Rad trans-Blot nitrocellulose membranes (Bio-Rad, Hercules, CA) for immunoblotting as described previously [45, 50].

### Ubiquitination assay

MCF-7 and MDA-MB-231 cells were co-transfected with MDM2 and ubiquitin plasmids, and treated with various concentrations of JapA as indicated in the figures. Cell lysates were immunoprecipitated with anti-MDM2 antibody, and the bound proteins were purified with protein G-Sepharose beads (Sigma, St Louis, MO), resolved on SDS-PAGE, and detected by an anti-ubiquitin antibody [45, 49].

### Immunofluorescence

MCF-7 and MDA-MB-231 cells were seeded on coverslips in a 12-well plate at a density of 10,000 cells/well, allowed to attach overnight, and treated with JapA (1 or 2 μM) for 24 h. The cells were fixed in a mixture of acetone and methanol (1:1), blocked in goat serum, and incubated with primary antibodies (anti-human MDM2 and β-actin antibodies) at 4°C overnight. Then, the cells were washed with PBS and incubated with Alexa Fluor 594 (anti-rabbit) and Alexa Fluor 488 (anti-mouse) with gentle shaking for 1 h, followed by DAPI nuclear counterstaining. The coverslips were mounted on slides and photographed under a fluorescence microscope (Olympus America Inc. Irving, TX) [54].

### Xenograft models and treatment

The animal study protocols were approved by the Institutional Animal Use and Care Committee of the Texas Tech University Health Sciences Center. Female athymic pathogen-free nude mice (nu/nu, 4-6 weeks) were purchased from Charles River Laboratories (Wilmington, MA). To establish MCF-7 human breast cancer xenografts, each of the female nude mice was first implanted with a 60-day subcutaneous (s.c.) slow release estrogen pellet (SE-121, 1.7 mg 17β-estradiol/pellet; Innovative Research of America, Sarasota, FL). The next day, cultured MCF-7 cells harvested from confluent monolayer cultures were injected s.c. (5 × 10^6^ cells, total volume 0.2 mL) into the left inguinal area of the mice [45, 50]. For the MDA-MB-231 xenograft model, we used the same procedure as above, but without the estrogen pellet. All animals were monitored for activity, physical condition, body weight, and tumor growth. The mice bearing MCF-7 and MDA-MB-231 xenografts were randomly divided into treatment and control groups (7-8 mice/group). The control group received the vehicle only. JapA was dissolved in PEG400:ethanol:saline (57.1:14.3:28.6, v/v/v), and was administered by intraperitoneal (i.p.) injection at doses of 15 or 30 mg/kg/d, 5 d/wk for 5 weeks (MCF-7) or 3 weeks (MDA-MB-231). The tumor mass (g) was calculated by the formula: (a×b^2^)/2, where “a” is the long diameter and “b” is the short diameter (cm). At the end of the experiments, the xenograft tumors, lungs, livers, kidneys, spleens and brains were removed from the mice, weighed, and snap-frozen for Western blotting, immunohistochemistry and hematoxylin and eosin staining. The breast tumor metastases to lungs were also counted.

### Hematoxylin and eosin (H&E) staining and immunohistochemistry

The hematoxylin and eosin staining was performed as described previously [55]. Briefly, freshly dissected tissues were fixed and embedded in paraffin. After being cut into 4-μm slices, the sections were deparaffinized and stained in Mayer's Hematoxylin and Eosin solution. Finally, the sections were dehydrated and mounted with Permount in a fume hood. The results were analyzed under a phase-contrast Olympus microscope (Olympus America Inc). For the immunohistochemical studies, the freshly dissected tissue was fixed in 10% neutral buffered formalin for 24-48 h. The tissue block was embedded in paraffin and cut to the desired thickness using a microtome, and was affixed onto a slide. After several wash cycles, the tumor sections were blocked and incubated with an anti-human MDM2 antibody (diluted 1:200 in 5% horse serum in PBS) overnight at 4°C. Subsequently, sections were incubated with pre-diluted streptavidin-peroxidase HRP conjugates in a humidified chamber at room temperature using a staining kit, according to the manufacturer's instructions (Dako North America, Inc., CA). The sections were counterstained with hematoxylin for 2-3 minutes and mounted and analyzed under a phase-contrast Olympus microscope (Olympus America Inc).

### Statistical analysis

The data were analyzed using the Prism software program version 6 (Graph Pad software Inc., San Diego, CA, USA). Student's *t-*test was used for comparisons between two groups. The quantitative data are reported as the means ± SEM from at least three independent experiments. Differences were considered to be statistically significant at *P* < 0.05. All statistical tests were two-sided.
